# Risk factors for insufficient reduction after short-segment posterior fixation for thoracolumbar burst fractures: Does the interval from injury onset to surgery affect reduction of fractured vertebrae?

**DOI:** 10.1186/s13018-022-03396-8

**Published:** 2022-11-24

**Authors:** Hiroyuki Aono, Shota Takenaka, Akinori Okuda, Takeshi Kikuchi, Hiroshi Takeshita, Keiji Nagata, Yasuo Ito

**Affiliations:** 1 Department of Orthopedic Surgery, National Hospital Organization, Osaka National Hospital, 2-1-14 Hoenzaka Chuo-ku, Osaka, Osaka 5400006 Japan; 2 Department Orthopedic Surgery, Osaka University Graduate School of Medicine, 2-15, Yamadaoka, Suita, Osaka 5650871 Japan; 3 Department of Orthopedic Surgery, Nara Medical University Hospital, 840, Shijocho, Kashihara, Nara 6348522 Japan; 4 Department Orthopedic Surgery, Kobe Red Cross Hospital, 1-3-1 Wakihamakaigandori, Chuo-ku, Kobe, Hyogo 6510073 Japan; 5 Department of Orthopedic Surgery, Saiseikai Shiga Hospital, 2-4-1 Ohashi Ritto, Shiga, 5203046 Japan; 6 Department Orthopedic Surgery, Wakayama Medical University Hospital, 811-1, Kimiidera, Wakayama, Wakayama 6418509 Japan

**Keywords:** Thoracolumbar burst fractures, Short-segment posterior fixation, Fracture reduction, Delay of surgery, Risk factor, Duration from injury to surgery

## Abstract

**Background:**

Many surgeons have encountered patients who could not immediately undergo surgery to treat spinal fractures because they had associated injuries and/or because a complete diagnosis was delayed. For such patients, practitioners might assume that delays could mean that the eventual reduction would be insufficient. However, no report covered risk factors for insufficient reduction of fractured vertebra including duration from injury onset to surgery. The purpose of this study is to investigate the risk factors for insufficient reduction after short-segment fixation of thoracolumbar burst fractures.

**Methods:**

Our multicenter study included 253 patients who sustained a single thoracolumbar burst fracture and underwent short-segment fixation. We measured the local vertebral body angle (VBA) on roentgenograms, before and after surgery, and then calculated the reduction angle and reduction rate of the fractured vertebra by using the following formula: $\left[ {\left( {{\text{Preoperative}}\;{\text{VBA }}{-}{\text{ Postoperative}}\;{\text{VBA}}} \right)/{\text{Preoperative}}\;{\text{VBA}}} \right] \, \times \, 100.$ A multiple logistical regression analysis was performed to identify risk factors for insufficient reduction. The factors that we evaluated were age, gender, affected spine level, time elapsed from injury to surgery, inclusion of vertebroplasty with surgery, load-sharing score (LSS), AO classification (type A or B), preoperative VBA, and the ratio of canal compromise before surgery.

**Results:**

There were 140 male and 113 female patients, with an average age of 43 years, and the mean time elapsed between injury and surgery was 3.8 days. The mean reduction angle was 12°, and the mean reduction rate was 76%. The mean LSS was 6.4 points. Multiple linear regression analysis revealed that a higher LSS, a larger preoperative VBA, a younger age, and being female disposed patients to having a larger reduction angle and reduction rate. The time elapsed from injury to surgery had no relation to the quality of fracture reduction in the acute period.

**Conclusions:**

Our findings indicate that if there is no neurologic deficit, we might not need to hurry surgical reduction of fractured vertebrae in the acute phase.

## Introduction

Approximately 90% of all spine fractures occur at the thoracolumbar junction, and thoracolumbar burst fractures, accounting for 10% to 20% of all spine fractures, make up one of the most common categories of spine fractures that are treated surgically [1–3]. According to the Denis classification [4], these fractures are two- or three-column injuries.

Short-segment posterior spine fixation is useful for such fractures because it preserves segment motion, provides superior kyphosis correction via indirect reduction, and is less invasive than other procedures. Although it was reported [5–7] in the 1990s that the procedure failed, there are now many reports of good surgical outcomes of short-segment fixation [8–10], and the procedure has become accepted because it reduces the deformity of the vertebral body and maintains the fracture reduction without major correction loss.

Because thoracolumbar burst fractures are high-energy injuries, patients with such fractures often have related injuries, such as head and/or abdominal injuries. For these patients, fracture reduction may be delayed by the presence of the concurrent injuries that must be treated first. Therefore, in the study we report here, we investigated the risk factors for insufficient fracture reduction after short-segment fixation of thoracolumbar burst fractures.

## Methods

Our inclusion criteria are single thoracolumbar burst fracture with AO type A3, A4, B1, and B2 with/without neurological deficit, treated by short-segment fixation, by ligamentotaxis, of the vertebra above the injured one and the one below it between September 2006 and August 2021 in five institutions. We excluded patients with AO type B3 and C, and multiple thoracolumbar burst fractures. Two hundred and fifty-three patients met these criteria. The protocol for our retrospective study was approved by the institutional review boards of all hospitals where the procedures took place, and all methods were carried out in accordance with relevant guidelines and regulations. The requirement for informed consent was waived because of its retrospective and observational study, and it is approved by institutional review boards.

The patients consisted of 140 males and 113 females with an average age of 43 years (range, 13 to 69 years). Their injuries were caused by falls from a significant height (169 patients), traffic accidents (64 patients), being hit by a falling object (9 patients), falling from horses (8 patients), and skiing accidents (3 patients). Thus, all sustained high-energy injuries. The level of spine involvement was T11 in 8 patients, T12 in 33, L1 in 110, L2 in 68, and L3 in 34.

### Outcome measures

Radiographic assessment was performed using supine anteroposterior and lateral roentgenograms and computed tomography (CT) scans before surgery. All patients were monitored radiographically by the use of standing or sitting anteroposterior and lateral roentgenograms and CT scans within 1 week after surgery. Five independent spine surgeons evaluated all radiographs and CT scans. The sagittal plane contour was assessed by measuring the vertebral body angle (VBA), which was the Cobb angle between the superior and inferior endplates of the injured vertebra, as we used in previous studies [9–11]. From these data, we calculated the reduction angle (preoperative VBA − postoperative VBA) and the reduction rate (%) using the following formula:

$$\left[ {\left( {{\text{Preoperative}}\;{\text{VBA }}{-}{\text{ Postoperative}}\;{\text{VBA}}} \right)/{\text{Preoperative}}\;{\text{VBA}}} \right] \, \times \, 100$$

**Fig. 1:**
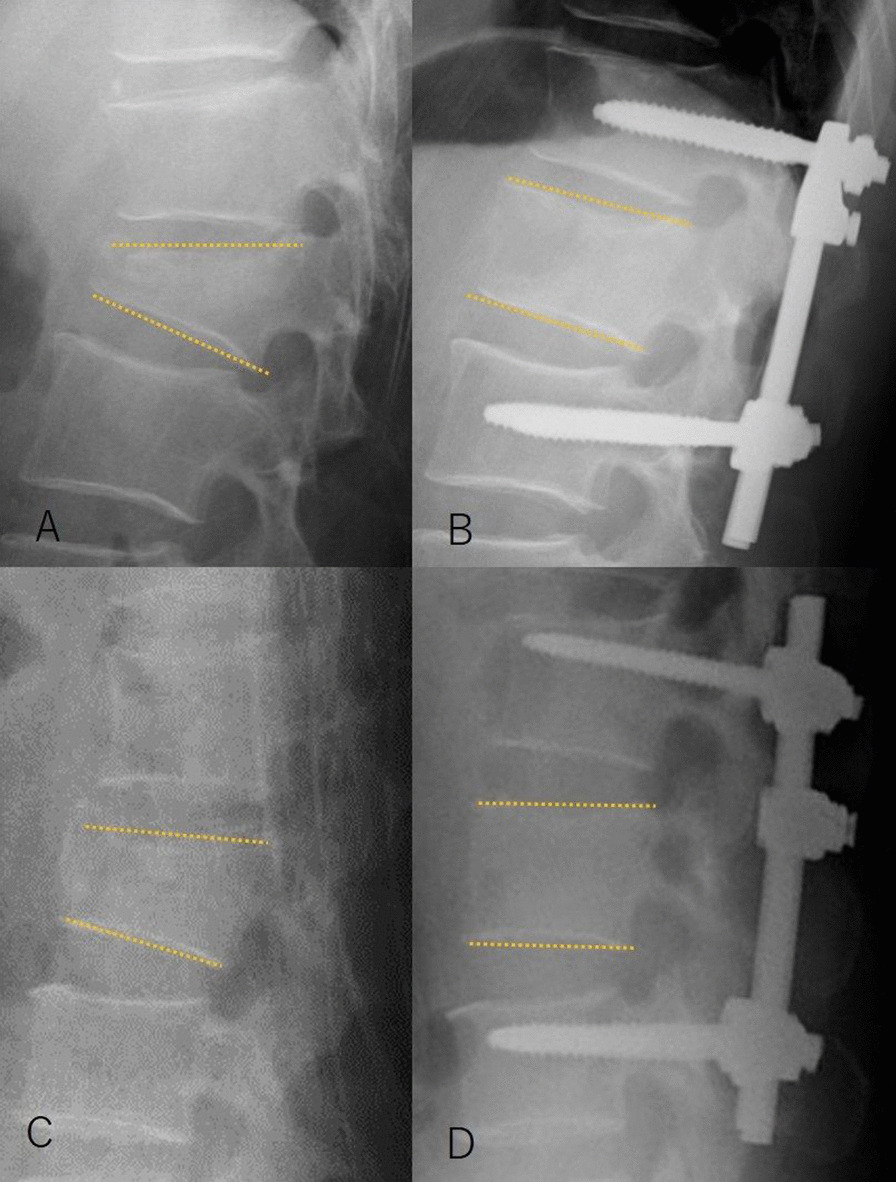
**a, b** Lateral radiographs from a 56-year-old man with an L1 burst fracture, showing changes before and after surgery. **a** The vertebral body angle (VBA) was 31° before surgery. **b** Surgery corrected the VBA to 3°. Therefore, the reduction angle was 28°, and the reduction rate was calculated as being 90% ([31 − 28/31] × 100). **c, d** Lateral radiographs from a 38-year-old man with an L1 burst fracture. **c** The VBA was 9° before surgery. **d** Surgery corrected the VBA to 0°. Therefore, the reduction angle was 9°, and the reduction rate was calculated as being 100% ([9 − 0/9] × 100). Thus, the reduction rate is suitable for patients with a minor deformity before surgery

When the reduction rate exceeded 100%, we counted that as 100% (Fig. 1).

Canal compromise was determined using CT scanning by directly measuring the anteroposterior canal dimension (in millimeters) at the maximum area of the retropulsed osseous fragment or fragments. This value was then compared with the average of similar dimensions measured above and below the injury. The result of this comparison was recorded as the canal compromise ratio (%) at the injured vertebra. Fracture severity was calculated using the load-sharing classification [6] and the AO classification [12].

### Surgical techniques

All surgical procedures were performed with the patients under controlled general anesthesia. Patients were placed in a prone position; initial postural reduction was then obtained. Pedicle screws were placed down into the pedicles of the bilateral vertebrae above and below the fracture. When we used Schanz pedicle screws, posterior wall decompression by indirect reduction via ligamentotaxis was performed, and for all 253 patients, segmental distraction by screws was performed. The surgical techniques used have been described in detail elsewhere [9, 10]. We performed additional vertebroplasty in 116 patients (46%). For fixation, we used Schanz pedicle screws (AO Universal Spine System, DePuy Synthes, West Chester, PA, USA) in 203 patients, the CD Horizon Longitude fixation system (Medtronic Sofamor Danek, Memphis, TN, USA) in 33, and the ES2 spinal system (Stryker, Kalamazoo, MI, USA) in 17.

### Statistical analysis

We used SPSS statistical software (version 21.0; IBM, Armonk, NY, USA) for all analyses; statistical significance was set at a *p* value of < 0.05. Group comparisons were conducted using Welch’s exact test for dichotomous variables. The correlation coefficient between two continuous or ordinal variables was analyzed using Spearman’s rank correlation coefficient test. Guilford [13] describes correlation coefficients of < 0.20 as being interpreted as “slight, almost negligible relationships”; correlations of 0.20 to 0.40 as “low correlation”; of 0.40 to 0.70 as “moderate correlation”; of 0.70 to 0.90 as “high correlation, marked relationship”; and of > 0.90 as “very high correlation, very dependable relationship.” To determine predictors of insufficient reduction, which was measured as the reduction angle and the reduction rate, we performed multiple linear regression analyses with stepwise selection. Before performing those analyses, we confirmed that the correlation coefficient between any two independent variables was < 0.7.

The correlated factors studied were age, gender, time elapsed between injury and surgery, affected level (T11–L1 *vs* L2 and L3), combination of vertebroplasty with surgery, AO classification (types A3 and A4 *vs* type B), LSS, preoperative VBA, and the ratio of canal compromise before surgery.

## Results

**Fig. 2:**
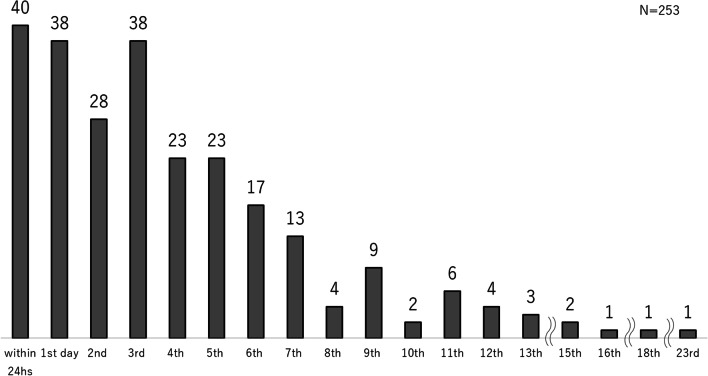
Distribution of elapsed time from injury until surgery for all patients

Forty patients were taken to an operating room for surgical stabilization within 24 h after injury. Another 38 patients underwent surgery within 48 h, and 89 patients had surgery within 3–5 days. The remaining 86 patients had surgery after 6 or more days (Fig. 2). Thus, the mean time elapsed between injury and surgery was 3.8 days (range 0–23 days).

### Radiological results

The VBA was corrected from 15.3° (range 38°–6°) before surgery to 3.8° (range 12° to − 5°) after surgery. The mean reduction angle was 11.5° (range 35°–3°), and the mean reduction rate was 76% (range 12–100%).

The mean ratio of canal compromise before surgery was 44% (range 5–89%). The mean score for fracture severity according to the LSS was 6.4 points. Nineteen patients had a score of 9 points, 57 had a score of 8, 60 had a score of 7, 63 had a score of 6, 35 had a score of 5, 13 had a score of 4, and 6 had a score of 3. Using the AO classification, we found that 89 patients had type A3 fractures, 75 had type A4, 24 had type B1, and 65 had type B2.

**Fig. 3:**
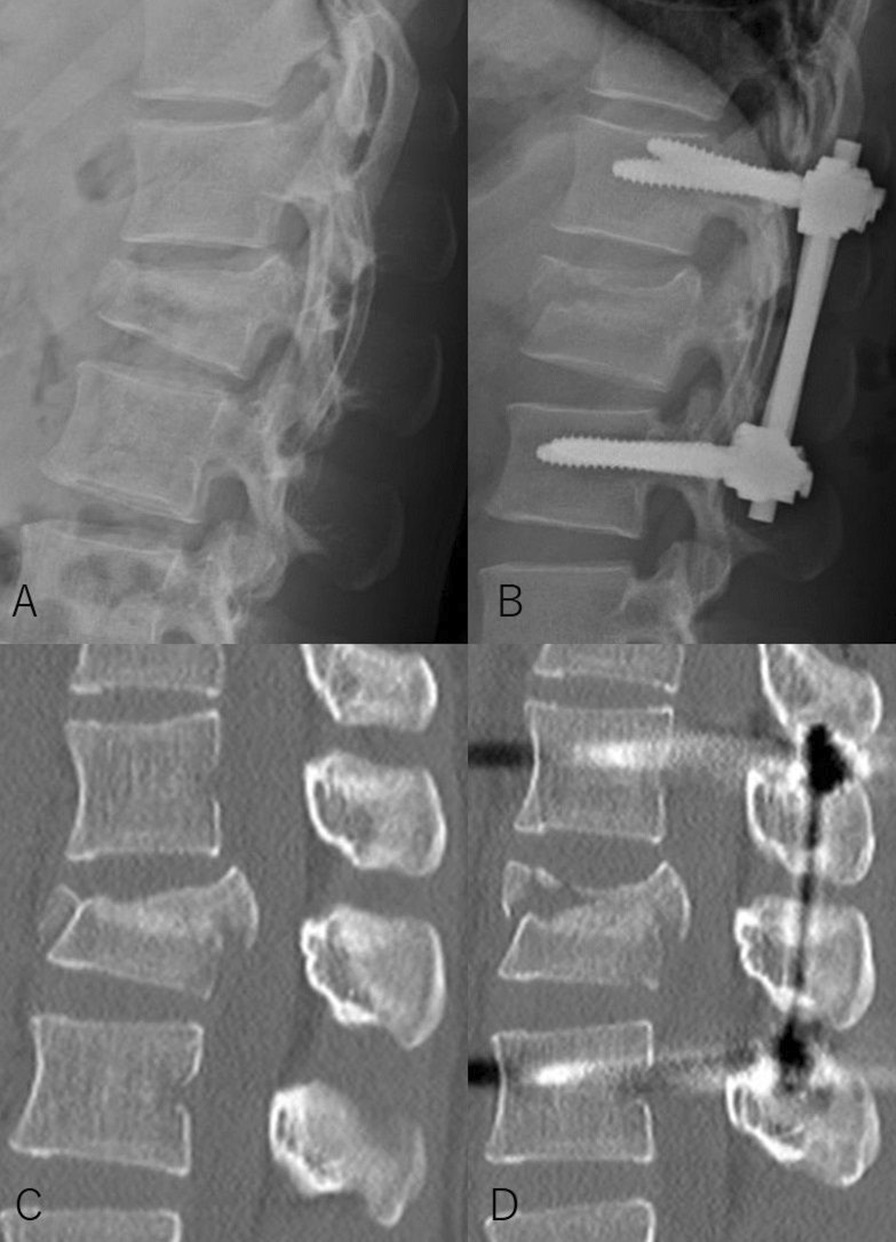
Lateral radiographs (**a, b**) and computed tomography (**c, d**) from a 19-year-old woman with an L1 burst fracture operated on 13th day after injury, showing changes before and after surgery. The vertebral body angle (VBA) was 21° before surgery. Surgery corrected the VBA to 4°. Thus, reduction of fractured vertebra is acceptable, though we could not perform surgery immediately

**Fig. 4:**
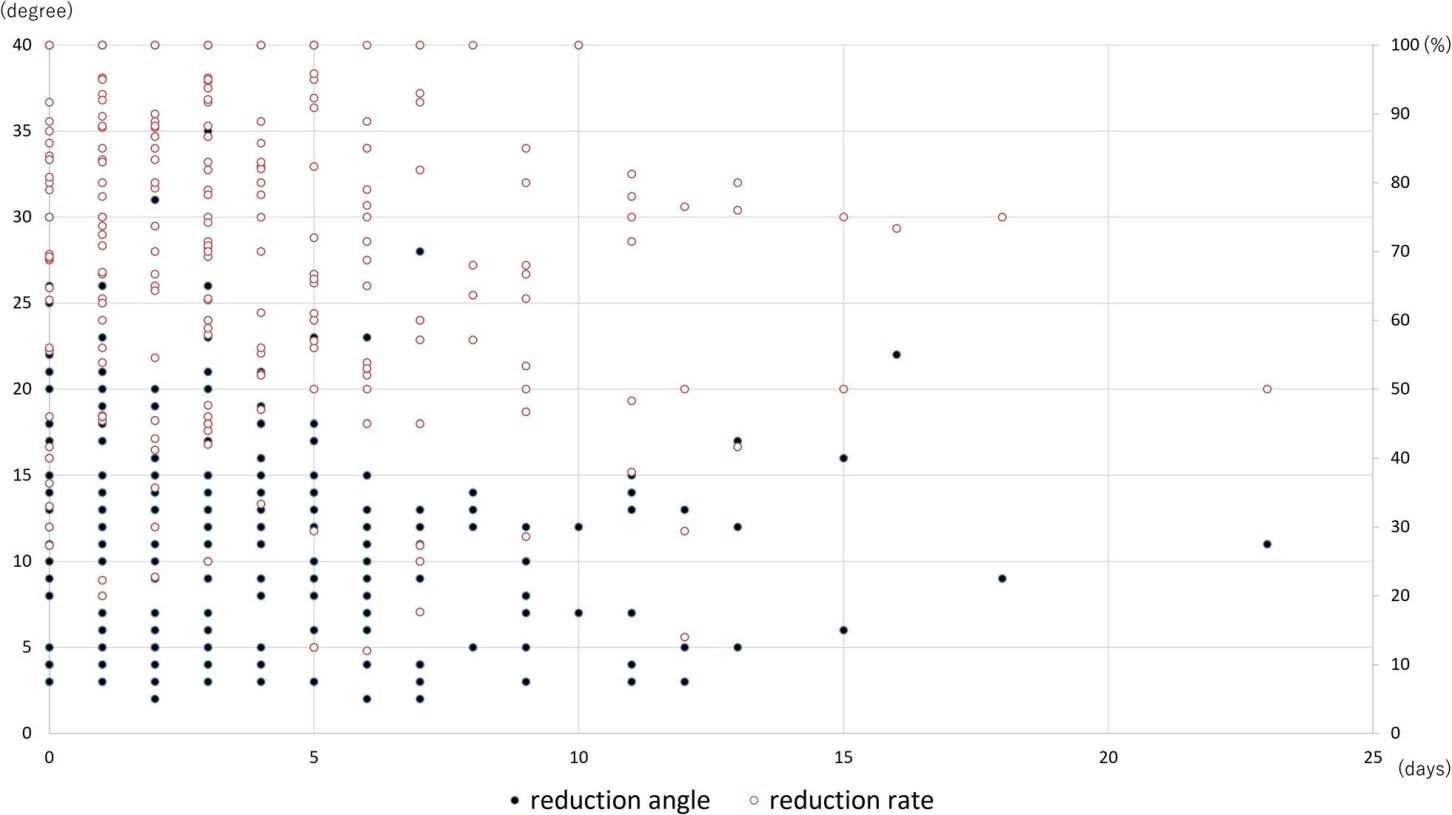
Correlation between duration from injury to surgery (day) and reduction angle/reduction rate. Duration from injury from surgery did not affect both reduction angle and reduction rate

Univariate analysis showed the following dichotomous factors to be significant regarding the reduction angle: gender and the AO classification (Table 1). In continuous and ordinal variables, age, preoperative VBA, and LSS had a moderate correlation with the reduction angle (Table 2). Regarding the reduction rate, gender was significant as a dichotomous factor (Table 1). There were no continuous and ordinal variables with a moderate or greater correlation with the reduction rate (Table 3). Multiple linear regression analysis revealed that a higher LSS, a larger preoperative VBA, a younger age, and being female make it more likely that patients will have a larger reduction angle and reduction rate (Table 3). The amount of time between injury onset and surgery did not affect either the reduction angle or the reduction rate (Figs. 3, 4).

**Table 1:** Univariate analysis for continuous and ordinal variables

Causal factors	*N*	ΔVBA (SD)	*p* value	Reduction rate (SDA)	*p* value
*Gender*					
Male	140	10.5 (6.6)		66.1 (28.8)	
Female	113	12.5 (6.9)	0.023^*^	76.2 (24.4)	0.003^*^
*Affected level*					
T10–L1	151	11.9 (6.7)		67.7 (25.5)	
L2 and L3	102	10.7 (6.8)	0.161^*^	74.8 (29.5)	0.050^*^
*Combination of vertebroplasty*					
Yes	115	12.1 (7.5)		74.2 (25.0)	
No	138	10.9 (6.0)	0.168^*^	67.6 (28.8)	0.052^*^
*AO classification*					
A3 and A4	164	10.7 (6.2)		70.1 (27.9)	
B1 and B2	89	12.7 (7.5)	0.039^*^	71.4 (26.4)	0.704^*^

*SD* standard deviation, *VBA* vertebral body angle

*Welch’s *t* test was used

**Table 2:** Correlation analysis between reduction angle and other continuous and ordinal variables

Parameter	*r*	*p* value
Age	− 0.407	< 0.001
Days since injury	− 0.210	< 0.001
Preoperative vertebral body angle	0.784	< 0.001
Preoperative canal compromise ratio	0.289	< 0.001
Load-sharing score	0.558	< 0.001

Spearman’s rank-order correlation test was used

**Table 3:** Multiple linear regression analyses

Parameter	Unstandardized		Standardized		*p* value
	*B*	SE	*β*	*t*	
*Reduction angle*					
Load-sharing score	1.076	0.154	0.256	6.969	< 0.001
Preoperative VBA	0.574	0.033	0.655	17.307	< 0.001
Age	− 0.045	0.15	− 0.113	− 3.075	0.002
Gender: male	− 1.926	0.466	− 0.142	− 4.132	< 0.001
*Reduction rate*					
Load-sharing score	7.165	1.034	0.423	6.929	< 0.001
Preoperative VBA	− 0.528	0.227	− 0.146	− 2.326	0.021
Age	− 0.294	0.098	− 0.183	− 3.001	0.003
Gender: male	− 9.424	3.132	− 0.172	− 3.009	0.003

*B*, unstandardized coefficients; *β*, standardized *B* coefficients; SE, standard error; *t*, *t*-value; and VBA, vertebral body angle

## Discussion

This study investigated the risk factors for insufficient fracture reduction after short-segment fixation of thoracolumbar burst fractures. As a result, a higher LSS, a larger preoperative VBA, a younger age, and being female were the factors that affected better reduction. It was not demonstrated that time elapsed from injury to surgery affected to the reduction of fractured vertebra in the acute phase.

Short-segment fixation is a widely accepted surgical procedure for thoracolumbar burst fractures, and there are many reports of success for this procedure with or without vertebroplasty [8–10]. Because this type of fracture involves high-energy injuries, patients with such fractures often have associated injuries. Aono et al. [9] reported that 81% of a group of these patients had associated injuries such as extremity fractures, pelvic fractures, and lung injuries. In patients with associated injuries, surgery cannot always be immediately performed because of their physical condition, and delays may result in insufficient reduction.

Jeon et al. [14] analyzed the factors affecting postural reduction in posterior surgery for thoracolumbar burst fractures. They found that a delay in performing surgery, the presence of a burst-split-type injury, and the presence of severe anterior vertebral compression were significant factors predisposing patients to insufficient postural reduction. In their 72-case series, they calculated the angular deformity of each fracture, including two adjacent disks (from the superior endplate of the vertebra one level above the fractured vertebra and to the inferior endplate of the vertebra one level below the fractured vertebra), including the fractured vertebra itself. When surgeons reduce the deformity of a fracture, they apply a reduction force not only on the fractured vertebra but also on the adjacent disks. Therefore, the data from Jeon et al. may be unreliable because it ended up including the angles of two adjacent disks. Moreover, they defined insufficient reduction as postoperative kyphotic deformity of > 20°, and 34% of their patients had insufficient reduction. Even if our measurement method is different, the mean postoperative kyphotic deformity was still 3.8°. Only 19 patients (8%) in our study had postoperative kyphotic deformity of > 10°, and no patient in our study had deformity of > 20°.

Our study had several limitations. First, it was based on radiological findings rather than on clinical findings. The risk factors we encountered in our study were present at the time of injury and thus could not be controlled. Further investigation will be required to determine which risk factors surgeons can control. Second, we did not use the same device to reduce and fix all fractures. In 203 patients (80%), we used Schanz screws and performed not only postural reduction and segmental reduction but also a lordosing maneuver using screws. However, for the remaining 50 patients, we performed only postural reduction and segmental distraction because the fixation device we used was not made for fracture reduction. However, Xu et al. [15] reported that postural reduction is effective in reducing thoracolumbar vertebral fractures, whereas instrumental reduction exerts only a relatively weak effect but is particularly useful for maintaining the results of postural reduction. Thus, the difference produced by the use of a device may not be an important factor. Third, we had a small number of patients for whom surgery had to be delayed. (Only 13% of the patients had to wait longer than 1 week for surgery.) That may be one of the reasons we could not determine a time limit for preventing insufficient reduction. However, it is natural and inevitable that the longer the period, the smaller the number of patients because of characteristics of injury. Further investigation with a larger number of patients who had delayed surgery may make it possible to determine a time limit.

The earlier surgery is done, the better. Some authors have reported that early surgical intervention reduces the overall complication rate in comparison with late surgery [16, 17]. However, patients with thoracolumbar burst fractures often have associated injuries that cause delays in surgical intervention. This is especially the case for patients with abdominal injuries, in which compression of the abdomen by use of the prone position is unsuitable, and for patients with head injuries, in which elevation of intracranial pressure by use of the prone position should be avoided. (We actually did have some of these patients.)

## Conclusions

In conclusion, patients with a higher LSS, a larger preoperative VBA, a younger age, and who were female had a better reduction angle and reduction rate for treatment of thoracolumbar burst fracture by short-segment fixation. Because it was not demonstrated in this study that the timing of surgery affected the results of fracture reduction, few delays are acceptable and we might not need to hurry such surgery at the point of fracture reduction in the acute phase, unless there was neurologic deficit.

## Data Availability

The datasets used and analyzed in the current study are available from the corresponding author on reasonable request.

## Abbreviations

CT: Computed tomography
VBA: Vertebral body angle
LSS: Load-sharing score
